# Scarcity-Coefficient Gated Projection Reinforcement Learning for Planning-Layer Capacity Activation in Emergency Wireless Networks

**DOI:** 10.3390/s26165248

**Published:** 2026-08-19

**Authors:** Jingxiang Ma, Ping Liu, Hongbin Ma, Guiping Lu, Youzhi Zhang

**Affiliations:** 1 School of Automation, Beijing Institute of Technology, Beijing 100081, China; 3220215147@bit.edu.cn (J.M.); 3120205436@bit.edu.cn (P.L.); 2 Marine Science and Technology Domain, Beijing Institute of Technology, Zhuhai 519088, China; lgpcan@bit.edu.cn; 3 Centre for Artificial Intelligence and Robotics, Hong Kong Institute of Science & Innovation, Chinese Academy of Sciences, Hong Kong, China; youzhi.zhang@cair-cas.org.hk

**Keywords:** planning-layer capacity activation, scarcity-coefficient gated projection reinforcement learning, regional upper-bound projection, constrained action decoding, capacity safety, emergency wireless networks

## Abstract

After infrastructure disruption, an emergency wireless controller must meet urgent communication demand and preserve resources for later periods. We propose Scarcity-Coefficient Gated Projection Reinforcement Learning (SCGP-RL). It jointly selects total planning-layer activation and a regional capacity upper-bound vector. Scarcity and urgent-demand evidence shape the activation intent. Scalar and capped-simplex projections enforce the coupled action constraints. A planning capacity unit (PCU) is defined as a calibratable service-capacity quantum. In the common constrained evaluation, SCGP-RL reduced the unmet urgent-demand score from 0.6643 for Projected CPO to 0.4919. It also satisfied all the executed hard constraints. Component tests show that the urgent-demand gate drives rapid service response and that the marginal demand-relief estimate provides a smaller benefit. Binding-condition tests show that the power, backhaul, and node-health mechanisms protect the resources they represent.

## 1. Introduction

Dynamic constrained resource activation arises when a controller must decide how much planning-layer capacity to activate before the future safety margin is fully known. Activating more capacity can satisfy time-sensitive demand in the current period, but it can also reduce the operating margin that is available to later periods; activating too little capacity protects the future while urgent communication opportunities expire. This sequential trade-off is central to emergency wireless restoration, where disrupted access, power, and backhaul support must still sustain command, shelter, and remote-reconnection communications [1,2]. Recent uncrewed aerial vehicle (UAV)-assisted emergency communication scheduling research further shows that temporary aerial support introduces fairness and scheduling constraints at the emergency-network layer [3]. The resulting planning-layer decision is dynamic capacity activation and regional capacity upper-bound allocation.

This decision differs from fixed-budget regional allocation because the total usable capacity is not given before the policy acts. Integrated access and backhaul (IAB) provides a useful high-level abstraction: a surviving donor connects to the core network while temporary nodes provide regional access and wireless backhaul support [4,5,6]. At the planning layer, changing power, backhaul, spectrum, and demand conditions determine how much capacity can be safely activated. A planning capacity unit (PCU) is a normalized service-capacity unit for one planning period rather than a standardized radio-resource unit. Technology-specific calibration translates regional PCU bounds into lower-layer resource or service limits. Activated PCUs expire at the end of each period, whereas energy and capacity risks persist across periods.

Existing studies address individual parts of this decision but rarely treat capacity activation and regional upper-bound formation as one action. Dynamic activation and sleep-control methods decide when infrastructure should be active or how aggressively resources should be admitted, but they often keep the regional allocation structure outside the activation decision [7,8]. Constrained Markov decision process (CMDP) and constrained reinforcement learning (CRL) methods, including constrained policy optimization (CPO), Lagrangian policy-gradient methods, CLARA-style resource allocation, and situational-constrained sequential resource allocation (SCRL), place costs or situation-specific limits into the learning objective or safety layer [9,10,11]. Projection and constrained action-decoding methods can repair fixed-budget regional allocations, while IAB-inspired resource allocation supplies access–backhaul capacity limits [12,13]. These lines of work leave open an action mechanism that learns the total activated capacity and the regional capacity upper-bound vector together.

To address this challenge, we propose Scarcity-Coefficient Gated Projection Reinforcement Learning (SCGP-RL). Its task-specific action factorization controls a state-dependent total activation amount and a regional upper-bound shape. Urgency, remaining-energy, and replenishment-risk evidence condition the scalar intent before projection. The decoder couples the scalar and vector components through $\sum_{i}x_{t,i}=B_{t}$. It preserves this equality after heterogeneous-energy rescaling. The method therefore extends fixed-budget projection by learning the simplex mass and its regional shape together. Standard PPO trains the policy over the resulting deterministic feasibility map.

The map-level formulation is grounded in analytic wargaming abstractions, where uncertain operational situations are organized as structured synthetic environments for examining decision consequences [14,15,16]. In this paper, that idea is used to build a computational regional planning map for the CMDP.

This paper makes three contributions.

1. We formulate the joint planning-layer action $\left(B_{t},x_{t}\right)$ and distinguish zero-residual per-period feasibility constraints from explicitly weighted soft planning-risk indicators.
2. We develop a compact nine-control action mechanism that couples scarcity-conditioned total activation with a deterministic regional score transformation, scalar projection, capped-simplex projection, and final joint energy rescaling.
3. We evaluate constrained performance, component effects, robustness, parameter sensitivity, and computational cost under a common experimental protocol.

## 2. Related Work

Dynamic activation and admission methods make the active resource amount a decision variable. Classical energy models and base-station sleep-mode studies show that activation state, sleep depth, and traffic-aware operation can dominate wireless energy use [7,17]. Sleep-mode surveys and recent energy-efficiency reviews further show that activation depth, load awareness, and operating mode selection remain central variables in green radio access networks [18,19]. Recent energy-saving and power-consumption studies extend this idea to learned or state-adaptive sleep control under traffic and supply uncertainty [8,20]. Recent reinforcement learning resource allocation in distributed LoRa networks provides another resource-activation example under energy-efficiency constraints [21]. Learning-assisted sleep-mode optimization provides another example in which a controller changes active capacity as part of the resource-allocation action [22]. These methods establish an admission-control view of how much infrastructure or load should be active. Their action spaces usually remain station-level, traffic-level, or link-level, whereas dynamic emergency capacity planning needs a scalar capacity action component that activates a period-level capacity amount and then forms regional capacity upper-bound vectors under future-risk constraints.

Constrained Markov decision process (CMDP) and constrained reinforcement learning (CRL) methods provide the formal basis for safety-aware sequential allocation. Constrained policy optimization (CPO) enforces expected cost constraints during policy improvement, and Lagrangian policy-gradient methods convert constraints into adaptive penalty terms [9,23]. Recent safe-RL studies emphasize state-wise feasibility and implementation-level safety constraints, which are important when admissible capacity varies with current resource state [24,25]. Safe-RL reviews and toolkits also emphasize that empirical safety evaluation should report utility and constraint indicators in addition to return [26,27]. Resource-allocation frameworks such as CLARA and situational-constrained sequential resource allocation (SCRL) show how resource budgets and situation-specific limits can enter the learning objective or feasibility logic [10,11]. Multi-agent deep reinforcement learning for UAV edge offloading and resource allocation similarly highlights state-dependent coupling among computation, energy, and communication resources [28]. These approaches establish the constraint-handling basis, but the activation amount and the regional upper-bound vector still need a task-specific action decoder.

Structured actions, scalar budget heads, and projection layers address how a policy produces feasible allocation actions. Differentiable optimization layers embed solver-like projections into neural policies, and constrained optimization learning studies how hard constraints can be respected by the prediction layer [29,30]. Projection onto the simplex or capped simplex is a standard operation for nonnegative fixed-sum allocation with upper bounds [12]. General continuous-control methods such as soft actor–critic and twin delayed deep deterministic policy gradients are natural candidates for scalar action heads, but they still require an external feasibility map when capacity bounds and regional caps change with state [31,32]. Work on joint resource allocation and trajectory planning in integrated sensing and communication (ISAC)-enabled UAV vehicular networks further illustrates how resource feasibility can be coupled with spatial motion decisions [33]. Capacity-upper-bound allocation adds a harder coupling because the fixed sum is itself part of the action. A direct continuous budget head may choose a scalar activation level, and a separate softmax or simplex head may distribute regional shares, but the two heads can remain inconsistent with energy, backhaul, and regional caps unless scalar projection and capped upper-bound projection are coupled.

Emergency wireless and access–backhaul resource sharing supply the capacity-risk variables behind these constraints. Disaster response studies emphasize communication continuity after conventional infrastructure and adjacent critical systems are impaired [1,2]. Integrated access and backhaul (IAB) research shows that access and backhaul share capacity and can be limited by donor-side backhaul limits, local devices, and temporary-node deployment [4,6]. IAB surveys clarify the access–backhaul coupling that motivates planning-layer backhaul caps, while recent deep reinforcement learning (DRL)-based IAB allocation studies illustrate the more detailed link-scheduling setting from which this paper abstracts [34,35]. Low-altitude platform and drone-base-station studies show that temporary node availability and aerial support can vary over time [36,37]. UAV-based emergency video-streaming research also shows that emergency response quality can depend on the joint availability of communication and edge resources [38]. Radio-frequency (RF) energy-supply and spectrum-management studies separately motivate planning-layer energy and spectrum-opportunity variables [39,40]. These works motivate urgent communication demand pressure, node health, spectrum opportunity, and backhaul caps as planning-layer state and constraint sources. The existing methods therefore establish dynamic admission, constraint learning, and projection-based feasibility, but they typically handle a fixed budget with regional allocation, a scalar admission action without regional upper-bound vectors, a training-objective constraint without a coupled decoder, or an access–backhaul scheduler with a predefined capacity model. A reinforcement learning action mechanism is still needed to learn total capacity activation and regional capacity upper-bound projection as one constrained planning decision.

CPO and adaptive Lagrangian methods control expected cost through policy updates, whereas fixed-budget projection forms a feasible regional vector from an externally supplied total. Direct projection can learn the total and regional controls jointly. SCGP-RL differs by using compact scarcity-conditioned controls to form both quantities before coupled projection. Its contribution is therefore the task-specific action mechanism rather than a new general-purpose constrained policy optimizer.

## 3. Task Modeling and Problem Formulation

### 3.1. Task Definition

After a disruptive event, emergency managers may have only a surviving access point and several temporary access–backhaul nodes to keep affected areas connected. These nodes provide planning-layer emergency-connectivity capacity, but the amount that can be safely activated in the current period changes with the remaining power margin, temporary backhaul load, and the urgency of nearby emergency communication demand. The central manager must therefore decide both how much planning-layer capacity to activate now and which regions can safely carry that activated capacity before detailed traffic scheduling begins.

The decision is difficult because present emergency communication demand and future capacity safety pull in different directions. Opening more capacity can help an urgent command post, shelter, or remote reconnection area before its urgent response period closes, but it can also consume operating margin that may be needed if replenishment is delayed or a backhaul path tightens later. Keeping capacity too conservative protects future periods but can let current high-priority communication demand go unmet. The same activation level can also be safe in one region and risky in another because local power margin, relay load, and urgent communication demand pressure differ across the affected area.

A PCU is defined by a planning period Δt and a reference useful-service quantum $Q_{\mathrm{ref}}$. Thus, $x_{t,i}$ PCUs represent an upper bound of $x_{t,i}Q_{\mathrm{ref}}$ useful bits or an average useful-rate bound of $x_{t,i}Q_{\mathrm{ref}}/\Delta t$. It is a planning variable rather than realized throughput. A lower-layer scheduler determines the delivered service under channel, interference, protocol, and user constraints.

If $W_{t,i}^{\mathrm{acc}}$ is usable access bandwidth, $\eta_{t,i}$ is effective spectral efficiency, and $C_{t,i}^{\mathrm{bh}}$ is available backhaul rate, the regional cap can be calibrated as

(1) $${\bar{x}}_{t,i}^{\mathrm{acc}}=\frac{\eta_{t,i}W_{t,i}^{\mathrm{acc}}\Delta t}{Q_{\mathrm{ref}}},\;{\bar{x}}_{t,i}^{\mathrm{bh}}=\frac{C_{t,i}^{\mathrm{bh}}\Delta t}{Q_{\mathrm{ref}}},\;{\bar{x}}_{t,i}=\min\left\{{\bar{x}}_{t,i}^{\mathrm{acc}},{\bar{x}}_{t,i}^{\mathrm{bh}},{\bar{x}}_{t,i}^{\mathrm{pow}},{\bar{x}}_{t,i}^{\mathrm{dev}}\right\}.$$

The power and device terms are converted to the same service-capacity scale. In LTE or 5G NR, $Q_{\mathrm{ref}}$ can be calibrated from usable resource-block time, spectral efficiency, protocol overhead, and scheduling duration. IAB adds donor and relay backhaul limits. Aerial systems also include air-to-ground availability and platform energy. The same interface can use technology-specific capacity models in future emergency-network deployments.

### 3.2. Regional Wargame-Style Planning-Map Modeling

**Figure 1:**
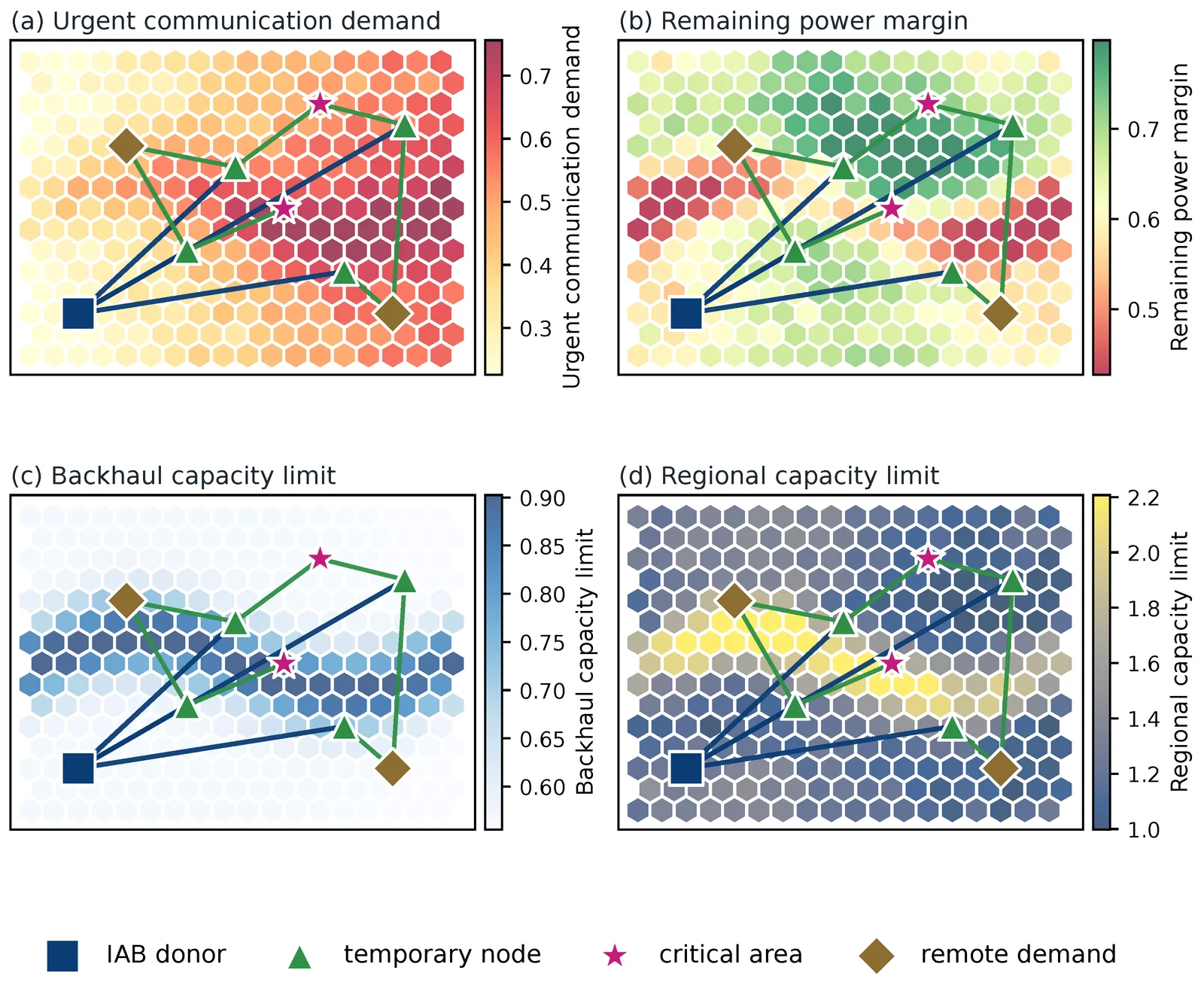
Regional emergency-planning map for dynamic capacity activation. The four panels encode (**a**) urgent emergency communication demand, (**b**) remaining power margin, (**c**) backhaul capacity limits, and (**d**) regional capacity limits. The shared marker legend below the panels identifies the surviving donor, temporary access–backhaul nodes, critical-communication areas, and remote-demand areas; blue and green edges denote backhaul and access-dependence links.

The regional map operationalizes the analytic wargaming abstraction as a computational planning object [14,15,16]. It exposes capacity activation trade-offs before lower-layer scheduling and defines the cells, overlays, and adjacency relations used by the CMDP; interactive adjudication and experiential play are outside this algorithmic model. To make the planning problem analyzable without reproducing every lower-layer link decision, the affected area is represented as a regional emergency-planning map. The map is tiled into regional cells; each cell denotes an emergency-connectivity support region or a temporary-node coverage area, and neighboring cells are connected by adjacency edges that represent possible emergency-traffic spillover, relay load, or access–backhaul dependence. The surviving donor and temporary-node markers in Figure 1 identify the access and relay resources available to the controller, while the critical-communication and remote-demand markers show why regional capacity cannot be interpreted only as local traffic volume.

The color overlays bind operational variables to the same regional cells. Figure 1a uses warm colors to mark urgent emergency communication demand, where an additional activated unit is most likely to reduce unmet urgent communication demand or accumulated backlog. Figure 1b maps remaining power margin, so low-margin regions can be separated from areas where extra activation is safer. Figure 1c maps backhaul capacity limits and highlights cells whose emergency-communication coverage depends on a constrained relay or donor path. Figure 1d maps the regional capacity limit, the largest planning-layer capacity upper bound that a region can safely offer in the current period.

The planning map therefore preserves the conflict between immediate emergency-demand relief and future capacity safety. A high-demand region in Figure 1a may still receive only a limited regional upper-bound vector when the power margin overlay in Figure 1b or the backhaul overlay in Figure 1c is tight. Conversely, a moderate-demand region can become important when its adjacency relation supports remote cells. The combined colors, markers, edges, and regional-limit overlay represent both sides of the capacity activation decision by showing when opening more capacity is worthwhile and which regions can carry that capacity without violating their caps.

### 3.3. Constrained MDP Formulation

The regional planning task is modeled as a constrained Markov decision process (CMDP) because current capacity activation changes later unmet emergency demand, energy margin, backhaul pressure, and feasible regional upper-bound vectors. At period *t*, the state $s_{t}$ contains regional demand and urgent communication demand pressure, accumulated unmet emergency demand, operating energy margin, replenishment forecast features, node availability and health, available spectrum, backhaul capacity, and regional capacity limits. Exogenous disturbances represent power recovery, temporary outage, node degradation, spectrum contraction, and backhaul degradation at the planning layer.

The action is

(2) $$a_{t}=\left(B_{t},x_{t}\right),$$

where $B_{t}$ is the executed total activated planning-layer capacity in the current period and $x_{t}=\left(x_{t,1},\ldots ,x_{t,N}\right)$ is the regional capacity upper-bound vector supplied as a regional upper bound to a downstream scheduler. The decoder implementation later distinguishes an intermediate scalar candidate $B_{t}^{\mathrm{cand}}$ from the post-filter executed value $B_{t}^{\mathrm{exec}}$; the CMDP notation writes the feasible executed total compactly as $B_{t}$. Let ${\bar{B}}_{t}$ denote the state-dependent total activation bound, and let ${\bar{x}}_{t,i}$ denote the tightened regional capacity limit after local node capability, power margin, spectrum opportunity, and backhaul capacity have been converted into planning-layer upper-bound caps. A feasible action must satisfy

(3) $$0\leq B_{t}\leq {\bar{B}}_{t},\;x_{t,i}\geq 0,\;\sum_{i}x_{t,i}=B_{t},\;x_{t,i}\leq {\bar{x}}_{t,i}.$$

When regional activation has heterogeneous energy cost $\epsilon_{t,i}^{E}$, the action also respects the operating-energy floor,

(4) $$\sum_{i}\epsilon_{t,i}^{E}x_{t,i}\leq \max\left\{0,E_{t}-E_{\min}\right\}.$$

The transition updates unmet emergency demand, regional pressure, operating energy, node availability, spectrum opportunity, and backhaul capacity after the capacity action is executed. A compact energy transition is

(5) $$E_{t+1}=\mathrm{clip}\left(E_{t}-\sum_{i}\epsilon_{t,i}^{E}x_{t,i}+H_{t+1},0,E_{\max}\right),$$

where $H_{t+1}$ is realized replenishment after the current action. PCUs expire at the end of the period, while remaining power, equipment condition, and future capacity opportunity persist across periods.

The constraints above define per-period feasibility and are enforced by the action decoder. The corresponding numerical residuals are reported in the experimental evaluation. Low power margin depth, service-window loss, demand debt, and future-capacity risk are normalized planning indicators used in the reward and evaluation.

The one-step reward balances immediate demand relief against activation cost, service loss, unmet-demand accumulation, and reserve risk. Let $R_{t}^{\mathrm{relief}}$ denote the reduction in demand risk produced by the executed action and let $C_{t}^{\mathrm{plan}}$ collect the normalized planning penalties. The policy objective is

(6) $$\max_{\pi_{\theta }}\;E_{\pi_{\theta }}\;\left[\sum_{t=0}^{T-1}\gamma^{t}r_{t}\right],\;r_{t}=R_{t}^{\mathrm{relief}}-C_{t}^{\mathrm{plan}},\;\left(B_{t},x_{t}\right)\in A\left(s_{t}\right).$$

The executable reward coefficients and normalization rules are reported in Section 5.2.

## 4. SCGP-RL

**Figure 2:**
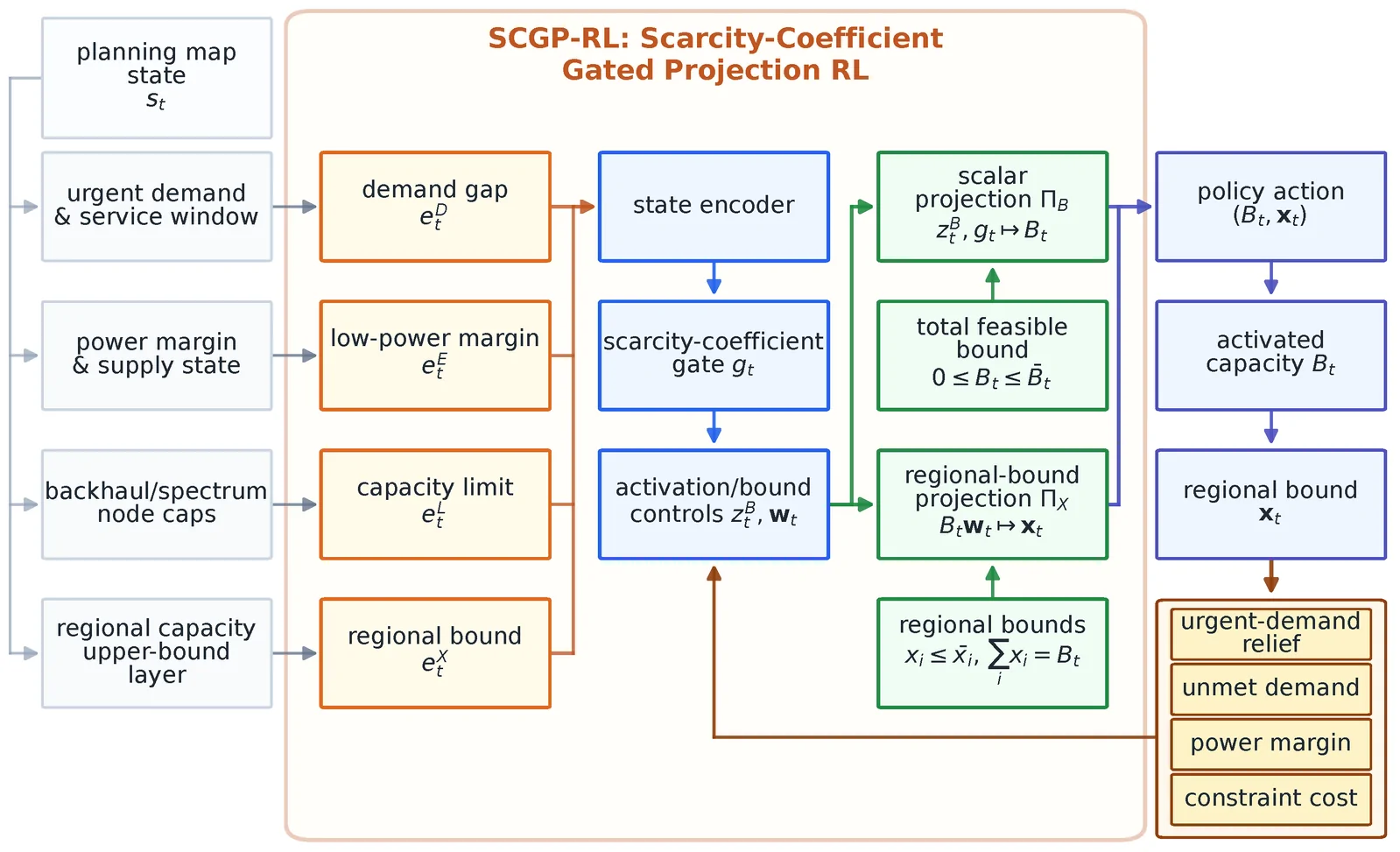
Scarcity-Coefficient Gated Projection Reinforcement Learning (SCGP-RL) action-generation core. The orange frame marks the proposed SCGP-RL mechanism, while planning-state inputs and executed-action feedback are external interfaces. Inside the core, capacity-risk evidence feeds the policy-control branch; the policy supplies scalar activation and upper-bound controls, so SCGP-RL generates the activated capacity as part of the action; the constrained decoder maps the scalar candidate and regional weights to the executed feasible capacity interface.

A feasible capacity action must determine both the total capacity activated in the current period and the regional upper-bound vector that bounds how this capacity can be consumed later. Fixed budgets remove the activation decision, and threshold rules react to only a small part of the urgent-demand and remaining-power trade-off. SCGP-RL turns capacity-risk state variables into urgent-demand, power, and backhaul risk evidence, lets the policy generate activation and upper-bound controls, and projects those controls into feasible capacity actions. The information flow in Figure 2 follows this mechanism from capacity, power, backhaul, and high-priority demand states to risk evidence and state-dependent feasible bounds. Policy controls then decide the total capacity activation intent and the regional upper-bound preference. A scalar projection forms an activation candidate, a regional upper-bound projection maps the preference into $x_{t}$, and the resulting feasible capacity action is executed before urgent-demand relief, unmet demand, and remaining power feedback return to the policy-improvement signal.

The method separates learned intent from feasibility enforcement. Urgent-demand, power, and backhaul risk evidence and the actor controls determine how much capacity the policy wants to activate and how it wants the regional upper-bound field to be shaped. Scalar and capped-simplex projections then act as deterministic constrained decoders that convert those controls into a feasible $\left(B_{t},x_{t}\right)$ action. This separation keeps the decoder as a deterministic feasibility map while the policy is evaluated through both utility and capacity safety feedback.

### 4.1. Capacity Availability and Risk Evidence

SCGP-RL begins by constructing the state-dependent feasibility set. The regional capacity limit ${\bar{x}}_{t,i}$ is the tightened planning-layer cap induced by local radio capability, available spectrum, node health, power margin, and backhaul support. The total activation bound is the capacity amount consistent with energy, device, spectrum, backhaul, and regional upper-bound limits:

(7) $${\bar{B}}_{t}=\min\left\{B_{t}^{E},B_{t}^{dev},B_{t}^{sp},B_{t}^{bh},\sum_{i}{\bar{x}}_{t,i}\right\}.$$

These bounds make the capacity action state dependent before the policy selects an activation level. Energy, spectrum, node-health, and backhaul limits therefore enter the action space through ${\bar{B}}_{t}$ and ${\bar{x}}_{t}$ as planning-layer feasibility terms before any lower-layer throughput translation.

Capacity-risk evidence describes why the policy should activate more or less capacity within that feasible set. Urgent communication demand pressure, accumulated unmet demand, and regional pressure indicate the cost of under-activation; remaining power margin, replenishment-risk features, node condition, and backhaul pressure indicate the cost of over-activation. We write this evidence compactly as

(8) $$e_{t}^{scar}=h^{scar}\left(s_{t},{\bar{B}}_{t},{\bar{x}}_{t}\right),$$

where $h^{scar}$ collects capacity availability, urgent communication demand pressure, and future-risk features already represented in the state.

The marginal urgent-demand-relief probe estimates whether a small additional capacity amount would reduce current communication demand pressure under the same local disturbance:

(9) $$\begin{array}{cc} M_{t}^{svc} & =\frac{J\left({\bar{s}}_{t+1}^{0}\right)-J\left({\bar{s}}_{t+1}^{\epsilon }\right)}{\epsilon },\;\;\; \end{array}$$

(10) $$\begin{array}{cc} M_{t}^{win} & =\frac{\Phi^{win}\left({\bar{s}}_{t+1}^{0}\right)-\Phi^{win}\left({\bar{s}}_{t+1}^{\epsilon }\right)}{\epsilon }. \end{array}$$

Here J(·) is the planning-layer communication demand pressure score, $\Phi^{win}\left(\cdot \right)$ is unmet urgent communication demand loss, and the two predicted next states compare zero additional activation with a small activation increment. The probe is part of the capacity-risk evidence used by the activation control.

### 4.2. Activation and Upper-Bound Controls

One policy actor produces nine controls. The first five regulate scarcity-conditioned activation, and the remaining four shape the regional upper-bound weights. Scarcity, gating, regional scoring, and projection are deterministic transformations of the state and policy controls rather than separate neural networks.

Let $M_{t}$, $p_{t}^{\mathrm{opp}}$, $p_{t}^{\mathrm{dem}}$, $p_{t}^{\mathrm{debt}}$, $p_{t}^{\mathrm{buf}}$, and $p_{t}^{\sup}$ denote the normalized marginal demand-relief estimate, service opportunity, demand pressure, debt, energy-buffer pressure, and replenishment-risk evidence. The executable scalar branch is

(11) $$\begin{array}{cc} V_{t}^{\mathrm{svc}} & =\mathrm{clip}(0.50M_{t}+0.20p_{t}^{\mathrm{opp}}+0.12p_{t}^{\mathrm{dem}}+0.06p_{t}^{\mathrm{debt}},0,1), \end{array}$$

(12) $$\begin{array}{cc} q_{t} & =V_{t}^{\mathrm{svc}}+\lambda_{t}^{\mathrm{win}}p_{t}^{\mathrm{debt}}-\lambda_{t}^{\mathrm{eng}}p_{t}^{\mathrm{buf}}-\lambda_{t}^{\mathrm{rep}}p_{t}^{\sup},\;\;\;\;\; \end{array}$$

(13) $$\begin{array}{cc} g_{t} & =\sigma (\alpha_{t}q_{t}+\beta_{t}).\;\;\;\;\;\;\;\;\;\;\;\;\;\; \end{array}$$

Thus the learned controls do not replace the deterministic evidence functions; they determine how strongly the current evidence opens or suppresses activation.

### 4.3. Coupled Projection and Policy Learning

This stage converts the policy controls into a feasible planning action. It first forms a bounded total activation candidate. It then constructs the regional preference vector and projects it onto the state-dependent capped simplex. A final energy filter rescales the scalar and vector components together. The resulting action is used for PPO learning. The activation candidate combines the gate output with a power-safety multiplier and an urgency safeguard:

(14) $${\tilde{B}}_{t}=E_{t}\min\left\{\rho_{t}^{\mathrm{feas}},\max\left(g_{t}s_{t}^{\mathrm{safe}},\rho_{t}^{\mathrm{floor}}\right)\right\},\;B_{t}^{\mathrm{cand}}={\mathrm{Proj}}_{\left[0,{\bar{B}}_{t}\right]}\left({\tilde{B}}_{t}\right).$$

Here, $s_{t}^{\mathrm{safe}}$ decreases activation as the reserve margin narrows, $\rho_{t}^{\mathrm{floor}}$ preserves a small urgency response, and $\rho_{t}^{\mathrm{feas}}$ represents the currently feasible activation ratio. The projection enforces the state-dependent total bound.

The implementation does not learn one neural score per region. A deterministic ROI/support function first constructs $r_{t,i}\geq 0$ from the observed regional features. Four actor outputs then form

(15) $$\begin{array}{cc} u_{t,i}^{\mathrm{ROI}} & =\frac{{\left[\max\left(r_{t,i}-\tau_{t}r_{t}^{\max},0\right)\right]}^{\gamma_{t}}}{\sum_{j}{\left[\max\left(r_{t,j}-\tau_{t}r_{t}^{\max},0\right)\right]}^{\gamma_{t}}+\varepsilon },\;\;\;\;\;\;\; \end{array}$$

(16) $$\begin{array}{cc} u_{t,i}^{\mathrm{soft}} & =\frac{\exp(\kappa_{t}r_{t,i})}{\sum_{j}\exp\left(\kappa_{t}r_{t,j}\right)},\;w_{t,i}=\left(1-m_{t}\right)u_{t,i}^{\mathrm{ROI}}+m_{t}u_{t,i}^{\mathrm{soft}}. \end{array}$$

The normalized vector $w_{t}$ is the desired regional upper-bound shape before feasibility correction.

The first regional step projects $B_{t}^{\mathrm{cand}}w_{t}$ onto the capped simplex:

(17) $$x_{t}^{\mathrm{cap}}={\mathrm{Proj}}_{X_{t}\left(B_{t}^{\mathrm{cand}}\right)}\left(B_{t}^{\mathrm{cand}}w_{t}\right),\;X_{t}\left(B_{t}^{\mathrm{cand}}\right)=\left\{x\geq 0:\sum_{i}x_{i}=B_{t}^{\mathrm{cand}},\;x_{i}\leq {\bar{x}}_{t,i}\right\}.$$

The projection is the Euclidean projection

(18) $${\mathrm{Proj}}_{X_{t}\left(B_{t}^{\mathrm{cand}}\right)}\left(y\right)=\arg\min_{x\in X_{t}\left(B_{t}^{\mathrm{cand}}\right)}{∥x-y∥}_{2}^{2}.$$

Because $B_{t}^{\mathrm{cand}}\leq \sum_{i}{\bar{x}}_{t,i}$, the set is nonempty and admits a water-level solution with $x_{i}=\min\left\{{\bar{x}}_{t,i},\max\left\{0,y_{i}-\tau \right\}\right\}$, where τ is chosen so that $\sum_{i}x_{i}=B_{t}^{\mathrm{cand}}$. The capped simplex uses the tightened regional caps defined above; when a spectrum, node-health, or backhaul limit is active, it has already reduced either the scalar bound or the relevant regional cap before this projection.

For heterogeneous energy costs, the executed action applies a final feasibility filter:

(19) $$\begin{array}{cc} \eta_{t} & =\min\left\{1,\frac{\max\{0,E_{t}-E_{\min}\}}{\sum_{i}\epsilon_{t,i}^{E}x_{t,i}^{\mathrm{cap}}+\varepsilon }\right\}, \end{array}$$

(20) $$\begin{array}{cc} B_{t}^{\mathrm{exec}} & =\eta_{t}B_{t}^{\mathrm{cand}},\;x_{t}=\eta_{t}x_{t}^{\mathrm{cap}}.\;\; \end{array}$$

When the energy bound has already been fully tightened, $\eta_{t}=1$; otherwise this filter rescales the scalar activation and the regional upper-bound vector as one action, preserving nonnegativity, regional caps, and the equality $\sum_{i}x_{t,i}=B_{t}^{\mathrm{exec}}$. Urgent-demand relief, unmet demand, and remaining power feedback enter the fixed shaped reward used by PPO; feasibility itself is provided by the decoder rather than by a constrained policy-gradient update.

SCGP-RL uses an actor and a reward–value function. The actor produces a distribution over the nine policy controls, and the critic estimates the scalar state value. The PPO update uses

(21) $$L^{\mathrm{clip}}\left(\theta \right)=E_{t}\;\left[\min\;\left(r_{t}\left(\theta \right){\hat{A}}_{t},\mathrm{clip}\left(r_{t}\left(\theta \right),1-\epsilon ,1+\epsilon \right){\hat{A}}_{t}\right)\right],$$

and combines the clipped policy loss with value and entropy terms. Network dimensions and optimization settings are reported in Section 5.2. Algorithm 1 summarizes the complete sequence from scarcity evidence construction to feasible action execution and policy feedback.
**Algorithm 1** Scarcity-Coefficient Gated Projection Reinforcement Learning (SCGP-RL) Capacity Activation Procedure.**Input:** State $s_{t}$, policy actor $\pi_{\theta }$, capacity-bound operators, scarcity evidence functions, and projection operators. **Output:** Executed feasible capacity action $\left(B_{t}^{\mathrm{exec}},x_{t}\right)$ and feedback on urgent-demand relief, unmet demand, and remaining power margin.
 1.**for** each decision period *t* **do** 2.Observe capacity, energy, backhaul, high-priority communication demand period, node-health, spectrum, and regional demand states. 3.Construct tightened regional caps ${\bar{x}}_{t,i}$ and the scalar activation bound ${\bar{B}}_{t}$ from the capacity-availability operators. 4.Build risk evidence $e_{t}^{scar}$ from communication demand pressure, remaining power pressure, replenishment risk, backhaul pressure, and the marginal urgent-demand-relief probe. 5.Select policy controls $\left(z_{t}^{act},z_{t}^{env},z_{t}^{risk}\right)=\pi_{\theta }\left(s_{t},e_{t}^{scar}\right)$ for total activation, regional upper-bound shape, and risk conservatism. 6.Transform the risk controls into scarcity coefficients and compute the gate score $q_{t}$ and activation gate $g_{t}$. 7.Combine $g_{t}$ with the safe-activation multiplier and feasibility-limited urgency floor to obtain ${\tilde{B}}_{t}$, and then apply scalar projection on $\left[0,{\bar{B}}_{t}\right]$ to obtain the scalar candidate $B_{t}^{\mathrm{cand}}$. 8.Score regional upper-bound preferences and normalize them into weights $w_{t}$. 9.Apply capped-simplex upper-bound projection on $X_{t}\left(B_{t}^{\mathrm{cand}}\right)$ to obtain cap-feasible regional upper bounds $x_{t}^{\mathrm{cap}}$.10.Apply the final energy-feasibility filter when heterogeneous unit costs require rescaling.11.Execute the planning-layer action $\left(B_{t}^{\mathrm{exec}},x_{t}\right)$ and expose $x_{t}$ as regional capacity caps to the downstream scheduler.12.Observe urgent-demand relief, unmet urgent communication demand loss, low power margin cost, and other constraint feedback.13.Update the policy with the PPO clipped objective and the reward in Equation (22).**end for**


The output of this layer remains a capacity formation interface upstream of downstream scheduling. The executed scalar component $B_{t}^{\mathrm{exec}}$ records how much planning-layer capacity is activated in the current period, and the vector $x_{t}$ records regional upper bounds for later use by task, user, or link-level decisions. When the final feasibility filter is inactive, $B_{t}^{\mathrm{exec}}=B_{t}^{\mathrm{cand}}$. This separation keeps SCGP-RL focused on dynamic capacity activation and regional capacity upper-bound allocation.

## 5. Experiments

The experiments address overall constrained performance, learned mechanism behavior, component contribution, stress robustness, parameter sensitivity, and deployment cost. Learned methods use independent training runs, validation-based checkpoint selection, and common final environments; paired inference preserves both training and environment variation.

### 5.1. Experimental Setup

The experimental environment uses a 15×18 regional emergency-planning map and a normalized emergency wireless simulator. Each episode contains time-varying communication demand pressure, energy replenishment, node availability, node health, spectrum availability, and IAB backhaul capacity. The scene in Figure 1 is used as a planning-layer map in which cells represent emergency-connectivity regions or temporary-node coverage areas, adjacency edges represent access or backhaul dependence, marker overlays identify donor, temporary, critical-communication, and remote-demand sites, and color shading indicates planning-layer communication demand pressure or capacity risk.

The canonical benchmark uses a 15×18 map, an 80-period horizon, $B_{\max}=8$, and energy values $E_{\max}=24$, $E_{0}=10.5$, and $E_{\min}=3.0$. Its replenishment process has normal, tight, reinforcement, and outage states with means (3.20,1.40,5.60,0.25), standard deviations (0.75,0.55,0.85,0.20), and transition matrix

$$P_{H}=\left[\begin{array}{cccc} 0.86 & 0.10 & 0.03 & 0.01\\ 0.18 & 0.72 & 0.04 & 0.06\\ 0.18 & 0.05 & 0.72 & 0.05\\ 0.34 & 0.30 & 0.04 & 0.32 \end{array}\right].$$

These values define the normalized planning scale. Section 5.8 varies the map, horizon, energy ratios, activation limit, and replenishment intensity. The implementation settings are summarized in Table 1, and the exact training, validation, and final-test seed sets are recorded in Table 2. Learned-policy intervals use crossed resampling over training and final-environment axes, and aligned comparisons use paired tests with Holm correction.

### 5.2. Implementation and Reproducibility

For the canonical map, the observation contains 31 global features and 14 features per region. The global features summarize episode phase, energy, replenishment, aggregate demand, service deficits, node condition, spectrum, and backhaul. The regional features describe demand, service continuity, node condition, radio and backhaul limits, capacity caps, energy cost, spatial position, and regional priority. This gives 3811 entries for 270 regions. Bounded state variables are normalized to [0,1].

The actor and reward critic use two 256-unit tanh layers. Their dimensions are 3811–256–256–9 and 3811–256–256–1, respectively. The actor parameterizes a nine-dimensional diagonal Gaussian. Its first five outputs control service-window scarcity, energy scarcity, replenishment risk, gate slope, and gate bias. The remaining outputs control the regional exponent, cutoff, softmax sharpness, and branch mixture. Their ranges are [0.12,1.22], [0.55,3.75], [0.35,3.00], [0.60,8.00], [−4.80,1.20], [0.20,8.00], [0,0.95], [0.10,12.00], and [0,0.85], respectively.

The executable reward is

(22) $$\begin{array}{cc} r_{t}= & \tanh\left(8{\tilde{G}}_{t}\right)-0.006B_{t}^{\mathrm{exec}}/E_{\max}-0.18\delta_{t}^{\mathrm{pol}}/E_{\min}-0.20L_{t+1}^{\mathrm{win}}-0.22D_{t+1}^{\mathrm{win}}\\ \; & -0.56C_{t+1}^{\mathrm{crit}}-0.16\delta_{t}^{\mathrm{risk}}/E_{\max}-0.10I\left(E_{t+1}\leq 10^{-6}\right)-0.08\Delta_{t}^{\mathrm{shock}}. \end{array}$$

Here, ${\tilde{G}}_{t}$ is the clipped demand-risk relief, $L_{t}^{\mathrm{win}}$ is service-window loss, $D_{t}^{\mathrm{win}}$ is demand debt, and $C_{t}^{\mathrm{crit}}=J_{t}+0.22L_{t}+0.20D_{t}+0.16{\left[L_{t}-0.155\right]}_{+}$. The reserve terms are normalized by the denominators shown above. The dynamic planning margin combines the hard reserve floor with demand pressure, replenishment risk, and the ${\mathrm{CVaR}}_{0.8}$ replenishment shortfall. The released source code provides the complete feature order and numerical implementation.

The batch-one deployment profile measures policy prediction and the complete decoder/environment decision path together with host and GPU memory use. SCGP-RL trains in 48.24 min for 120,000 steps. Mean policy-prediction latency is 0.425 ms, and the complete prediction–decoder–environment path takes 14.617 ms with a p95 of 14.833 ms. The measured training GPU-memory increment is 383 MiB, and deployment RSS is 795.4 MiB.

Let *N* be the number of regions, *P* the neural-parameter count, and *I* the fixed projection iteration count. One SCGP-RL decision has complexity $O(P+N^{2}+IN+N\log N)$. For *T* environment steps and *K* PPO epochs, training has complexity $O(KTP+T\left(N^{2}+IN+N\log N\right))$. The measured time and memory values are reported in Table 2.

The stress evaluation varies supply stability, initial reserve, urgent demand, backhaul capacity, spectrum availability, and node condition. These settings create nine scenarios that expose different resource bottlenecks, as summarized in Table 3. All the methods face the same environment realizations within each scenario.

### 5.3. Comparison Methods and Evaluation Metrics

The comparison set is organized by the decision logic each baseline uses. Always-on full-activation sets $B_{t}={\bar{B}}_{t}$, and uniform regional bound spreads the regional upper-bound vector evenly under the regional caps. Fixed-activation RL and fixed-activation rule both use $B_{t}=\min\left\{6.0,{\bar{B}}_{t}\right\}$; the learning version trains the regional upper-bound branch with the same clipped policy-gradient backbone used by the trainable baselines [23], while the rule version applies a fixed regional pattern. Target power margin activation uses $E_{t}^{🟉}=E_{\min}+\left(0.40-0.25{\bar{H}}_{t}/H_{\max}\right)E_{\max}$ and then $B_{t}=\min\left\{{\bar{B}}_{t},{\left[E_{t}-E_{t}^{🟉}\right]}_{+}/\epsilon_{t}^{max}\right\}$, where ${\bar{H}}_{t}/H_{\max}$ is the normalized replenishment forecast and $\epsilon_{t}^{max}=\max_{i}\epsilon_{t,i}^{E}$. Robust power margin activation adds a 0.12 coefficient on the replenishment coefficient of variation and a 0.10 coefficient on outage pressure to that minimum-margin rule, while high power margin rule uses $E_{t}^{🟉}=E_{\min}+0.48E_{\max}$. These power-margin and threshold references adapt energy-aware activation and sleep-control ideas to the planning layer [7,8]. Target power + regional bound and target power + urgent-demand bound incorporate regional value or near-deadline urgent-demand pressure into the activation ratio and regional upper-bound weights. Rolling-horizon marginal activation forms a one-step emergency-demand-relief value from 0.40 urgent-demand opportunity, 0.34 unmet-demand backlog pressure, and 0.26 demand pressure, modulated by remaining power pressure. Lyapunov drift-plus-penalty builds its heuristic action from high-priority demand pressure, replenishment risk, and remaining power queues, while the dual and queue-style references provide deterministic constrained-resource controls.

The implemented trainable projection references are Direct-Projected PPO and Conservative-Projection PPO. Direct-Projected PPO uses a direct scalar activation head with a softmax regional-bound feasibility interface. Conservative-Projection PPO uses ordinary PPO with a direct activation head, the common projection interface, a conservative activation ceiling of 0.90, and fixed reward penalties; it does not use a cost critic, cost budget, multiplier, or dual update. CPO, CLARA, and SCRL provide the constrained-learning background [9,10,11]. Projected CPO provides the direct constrained-RL baseline. It uses the same observation, training budget, evaluation protocol, executed action, and feasibility decoder as SCGP-RL. Its policy outputs one activation control and *N* regional logits. Its clipped nonnegative cost combines normalized reserve shortfall with weighted service-window loss, demand debt, loss exceedance, and energy depletion. The corresponding weights are 1, 0.22, 0.20, 0.16, and 1. The episode-mean cost budget is 0.45. Rule and planning baselines use current and history-derived information, while component contributions are evaluated through complete removals and evaluation-time disabling.

The reported metrics follow the wireless-capacity formulation. The unmet urgent-demand score is written as $C^{crit}=T^{-1}\sum_{t}\sum_{w}\omega_{w}\chi_{w}^{crit}\ell_{t,w}^{svc}$ and measures weighted unmet communication demand in high-priority periods. The unmet-demand backlog $\bar{D}=T^{-1}\sum_{t}D_{t}^{svc}$ measures unmet requirements carried into later periods, while the service-loss exceedance measures how far service-window loss exceeds 0.155. The low power margin period rate $v_{E}=T^{-1}\sum_{t}I\left(E_{t}<E_{t}^{min,plan}\right)$, mean remaining power, and remaining power margin quantify whether the policy stays above a dynamic planning-layer minimum power margin. The line $E_{t}^{min,plan}$ defines the planning-layer risk and reward penalty threshold; every policy also satisfies hard per-period activation feasibility through $B_{t}^{E}$. Unit-energy urgent-demand relief $u_{E}=\sum_{t}G_{t}^{svc}/\left(\sum_{t}\sum_{i}\epsilon_{t,i}^{E}x_{t,i}+\varepsilon \right)$, cumulative demand-relief gain, mean activated capacity, activated-capacity utilization, backhaul-capacity utilization, and remaining capacity headroom measure whether the policy activates capacity where it helps urgent communication while preserving future capacity. Because the evaluation intentionally keeps persistent demand pressure, the binary high-priority demand-period miss indicator tends to saturate; the interpretation therefore combines service-loss exceedance, unmet-demand backlog, unmet urgent-demand score, low power margin period rate, remaining power margin, and efficiency.

### 5.4. Overall Performance Comparison

**Figure 3:**
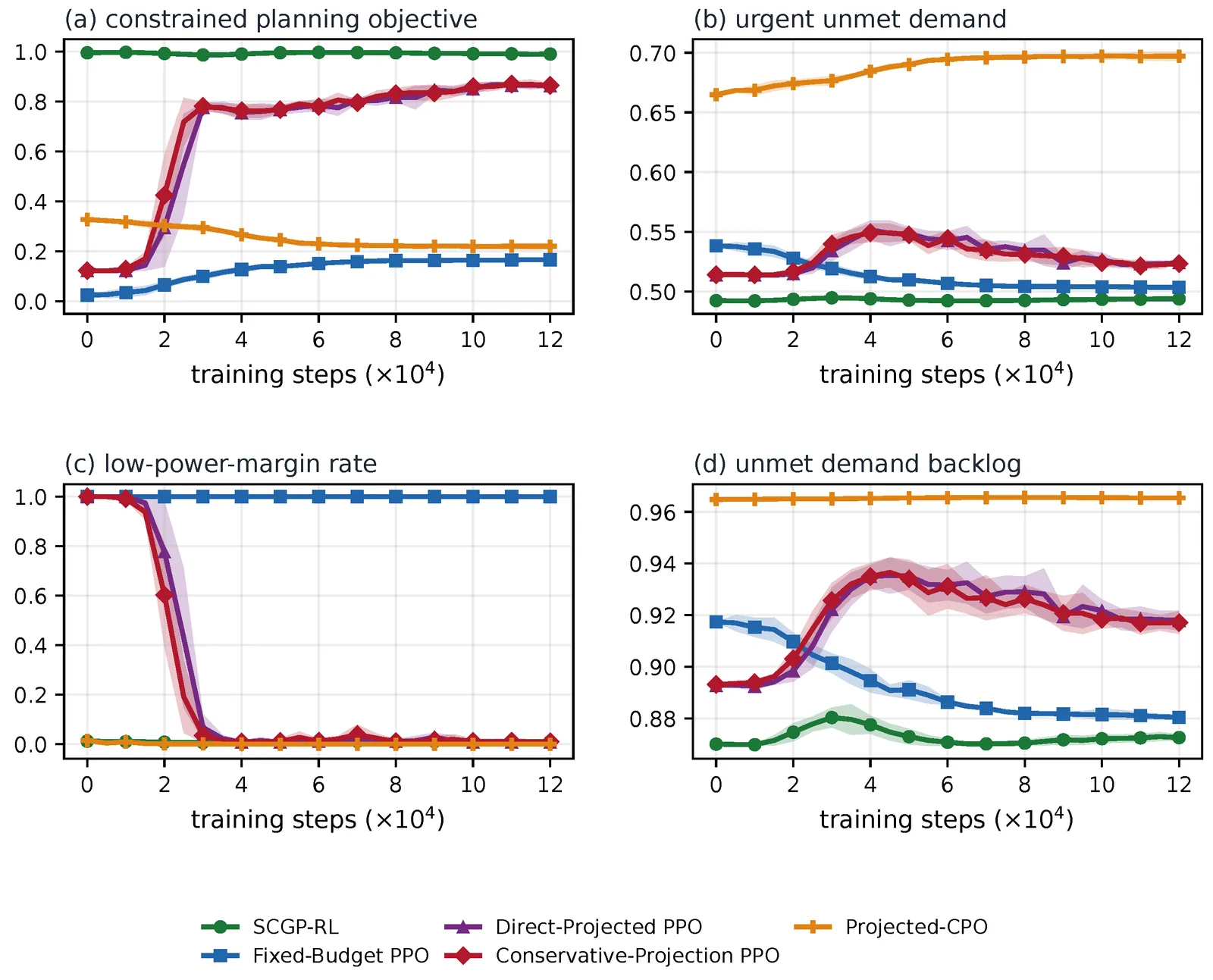
Validation learning curves used for checkpoint selection. Lines show the mean across training runs, and bands show one standard deviation.

**Figure 4:**
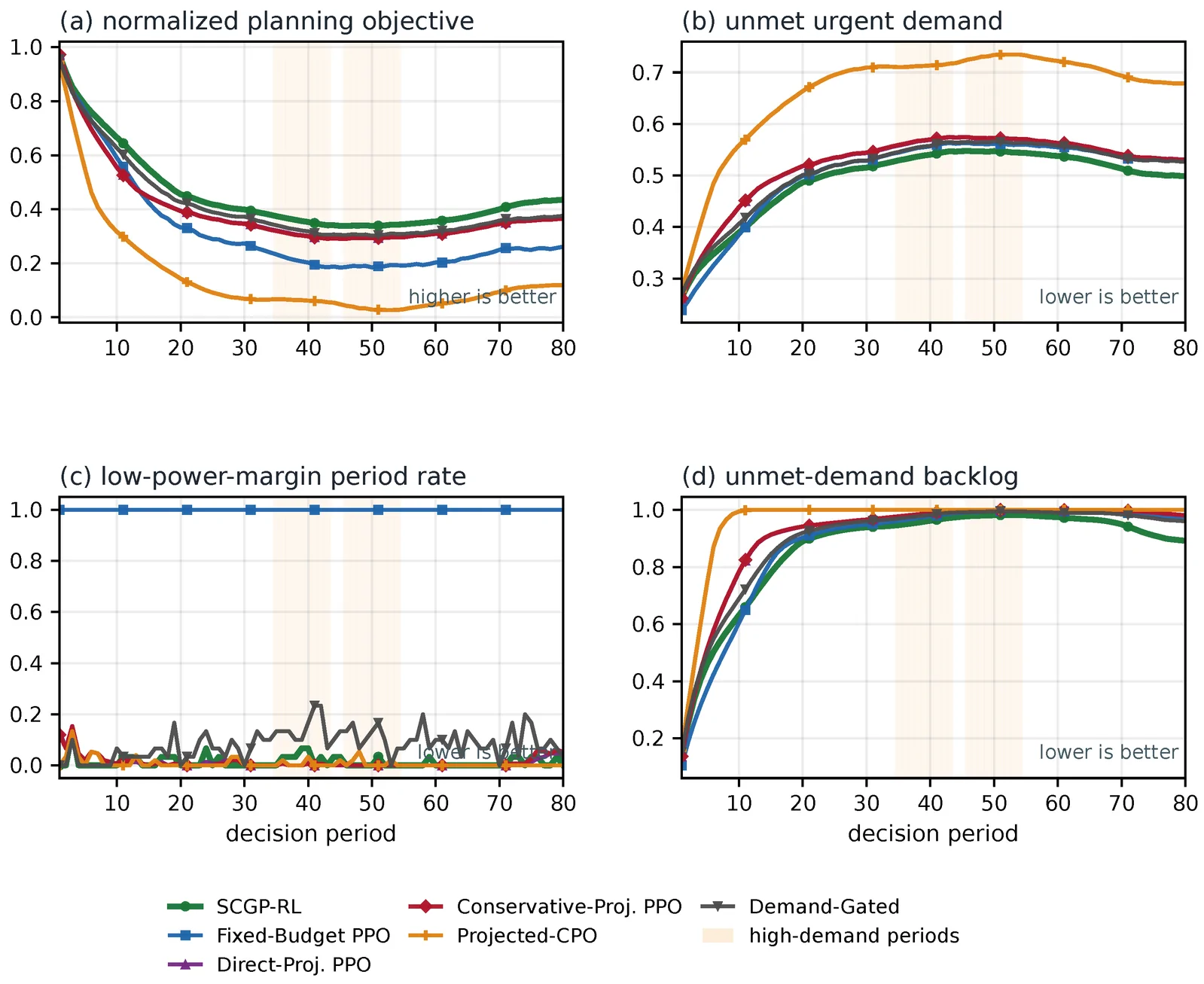
Time-resolved performance in the common held-out trajectories. The panels show the planning objective, unmet urgent demand, low power margin rate, and unmet-demand backlog. Shading marks high-demand periods.

The overall evaluation examines whether SCGP-RL improves urgent-demand service while maintaining the planning power margin and hard action feasibility. Independently trained policies and deterministic rules are evaluated in common held-out environments using unmet urgent demand, demand backlog, remaining power, activated capacity, and low power exposure. As shown in Table 4, SCGP-RL achieves the lowest unmet urgent-demand score while keeping low power exposure close to the most conservative learned policies. Its advantage over Projected CPO is confirmed by the paired analysis in Table 5. The learning curves in Figure 3 show stable checkpoint selection across training runs, and the time-resolved results in Figure 4 show that the gain is concentrated in high-demand periods rather than produced by uniformly increasing activation. The improvement therefore arises from when and where capacity is opened while the planning margin is preserved.

### 5.5. Learned Capacity Activation Behavior

**Figure 5:**
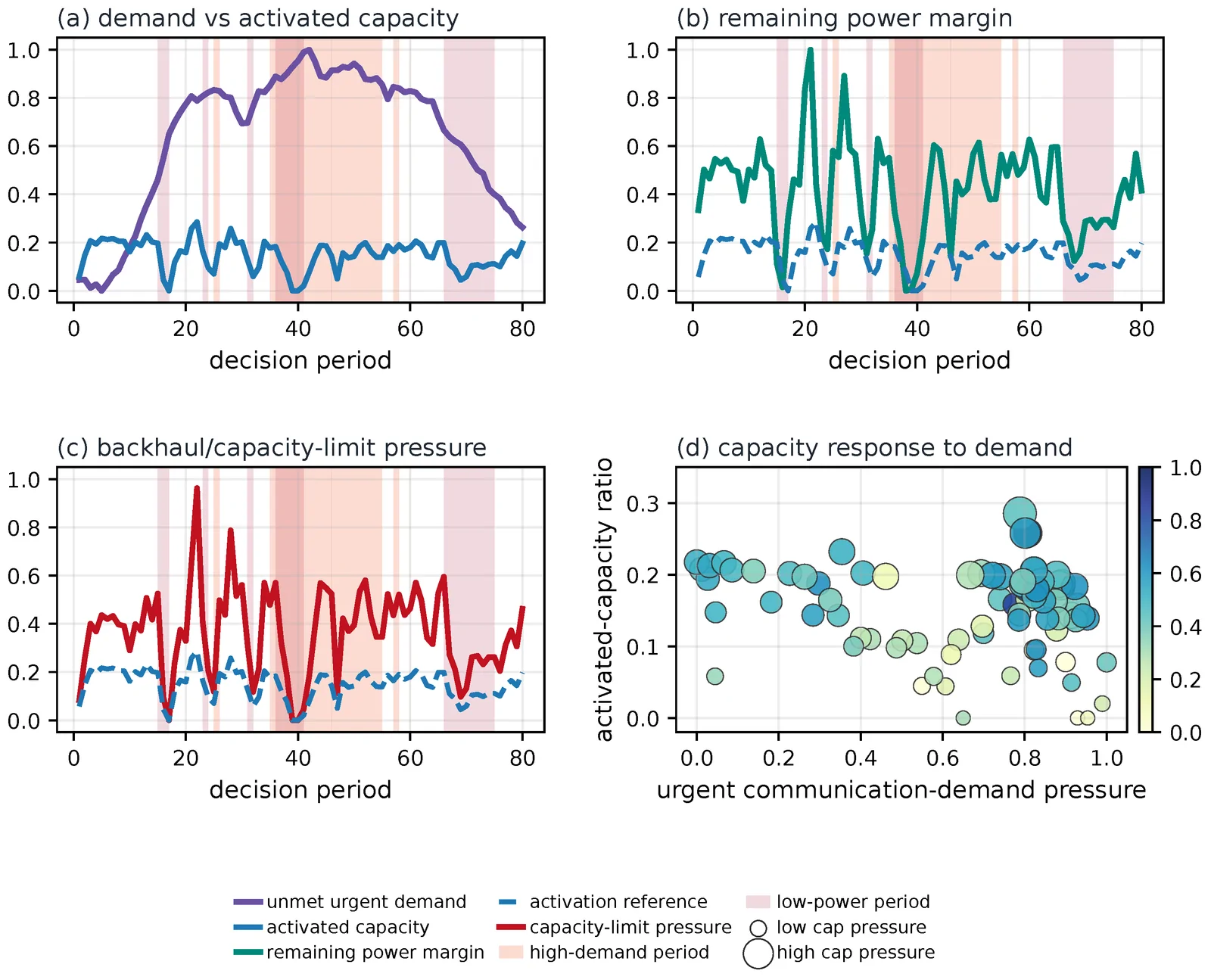
Representative SCGP-RL trajectory showing activation, urgent demand, remaining energy, and capacity-limit pressure.

**Figure 6:**
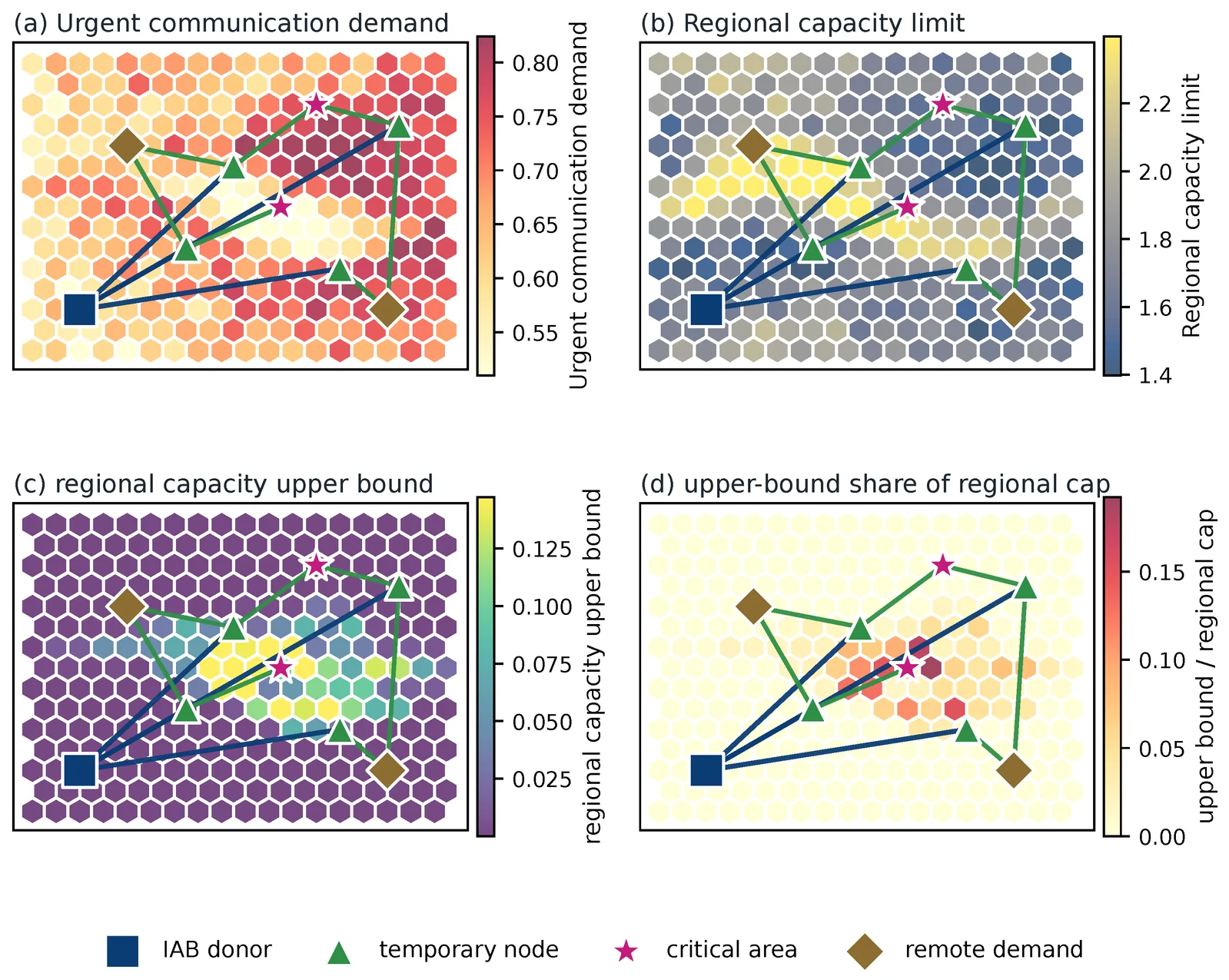
Spatial capacity-envelope example showing urgent demand, regional caps, allocated upper bounds, and cap utilization. Blue and green lines denote backhaul and access-dependence links, respectively.

**Figure 7:**
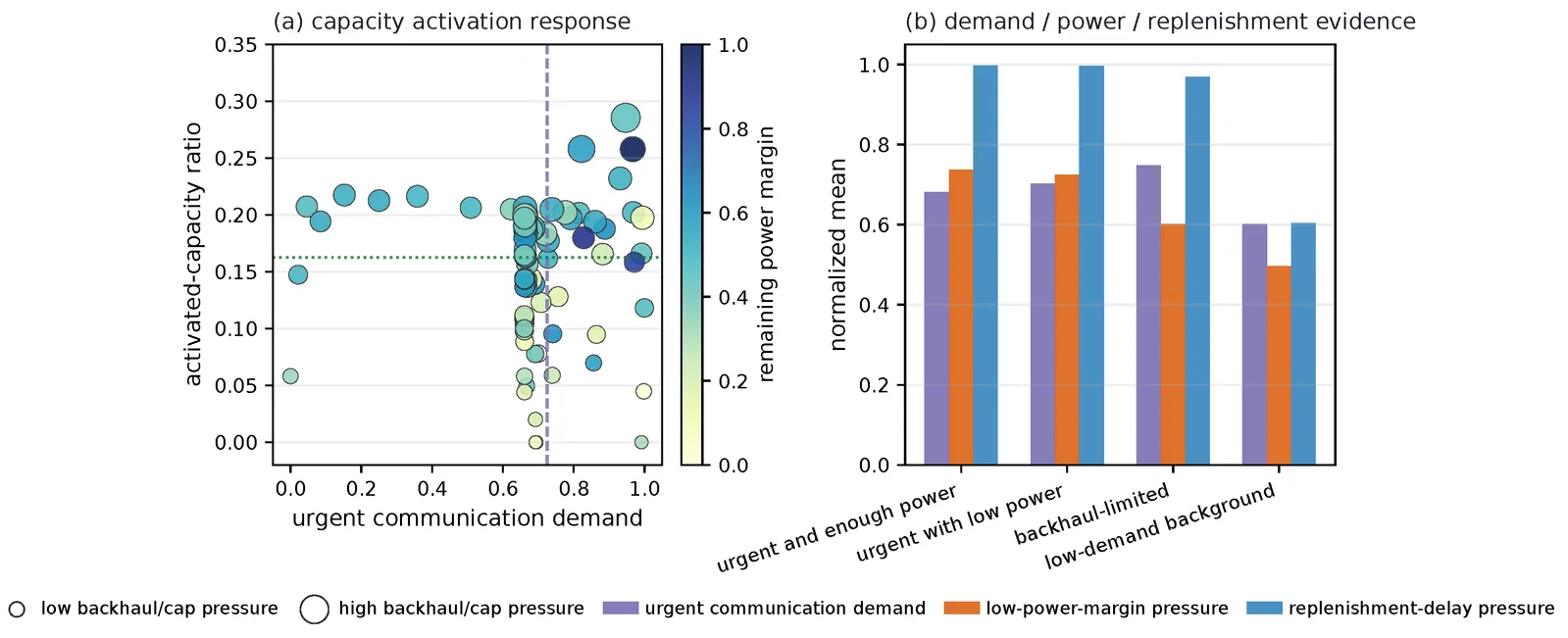
Activation behavior and normalized scarcity evidence across resource-risk regimes. In (**a**), the vertical dashed line marks the 68th percentile of the normalized urgent-demand signal, and the horizontal dotted line marks the median activated-capacity ratio.

The behavior analysis examines how demand and resource scarcity shape the learned action. A representative trajectory is selected by its proximity to the median unmet urgent-demand score. Figure 5 shows that the policy limits activation when energy or backhaul becomes scarce and permits larger regional bounds when the operating margin improves. The spatial case in Figure 6 further shows that the regional upper-bound field is localized relative to communication pressure and local capacity limits. A higher value allows the downstream scheduler to use more planning capacity in the corresponding region. The regime summary in Figure 7 links these decisions to the normalized scarcity evidence and separates urgent but safe periods from energy-risk and backhaul-tight periods.

### 5.6. Component Contribution Analysis

The component study separates adaptive effects from immediate mechanism use. Component-removal policies show whether the remaining controls compensate during learning, while direct disabling isolates the decision effect under the same policy. Table 6 shows that adaptation offsets much of the aggregate impact of removing a demand-guidance component, whereas removing power margin planning increases service-loss exceedance and reduces demand-relief gain. Table 7 shows that the urgent-demand gate is the main demand-response pathway. The marginal demand-relief estimate has a smaller effect, and the urgency safeguard remains inactive in the evaluated trajectories. All the variants retain the common physical execution constraints.

### 5.7. Robustness Under Resource Stress

**Figure 8:**
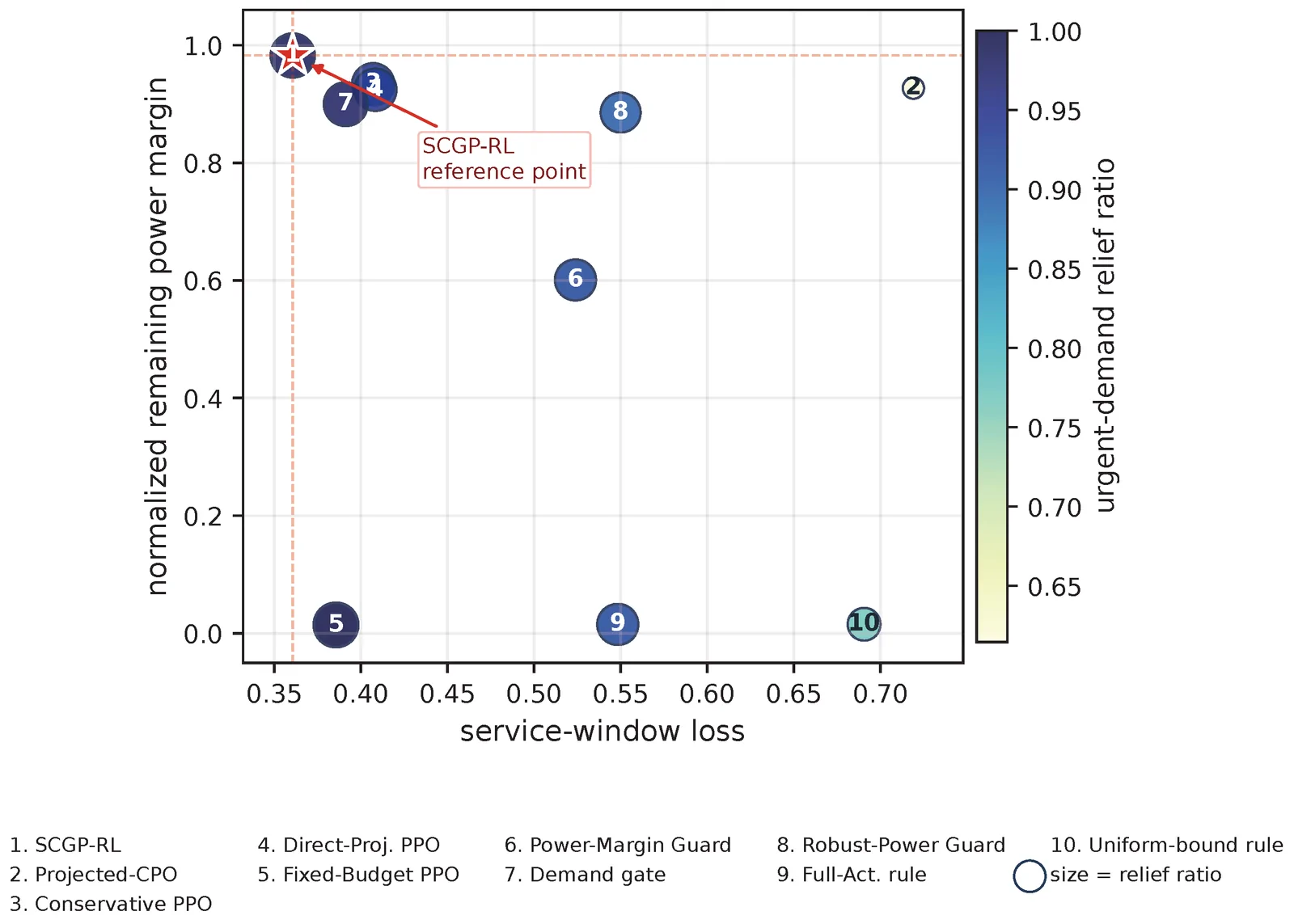
Energy and urgent-demand trade-off across the stress scenarios. Bubble size and color encode the urgent-demand relief ratio. The star marks the SCGP-RL reference point, and the dashed lines show its coordinate projections.

The robustness study tests whether the learned capacity policy preserves demand service and resource margins when the dominant bottleneck changes. Nine scenarios vary supply stability, urgent demand, backhaul availability, spectrum availability, and node condition. Table 8 shows that SCGP-RL maintains low service-window loss and low power exposure while retaining near-best demand relief. Figure 8 places it in a favorable region of the service–energy trade-off. Binding-condition tests explain this behavior: removing power margin planning increases reserve shortfall under severe scarcity, while removing the backhaul cap or node-health mechanism produces excess proposals when the corresponding physical limit becomes active.

### 5.8. Parameter and Map-Size Sensitivity

The sensitivity study examines whether the conclusions depend on individual configuration choices or the planning-map scale. Initial energy, reserve floor, decision horizon, activation ceiling, and replenishment intensity are varied one at a time around the canonical setting. Table 9 shows that SCGP-RL retains its service advantage across these changes, while resource indicators respond in the expected direction to tighter energy conditions. The map-size experiment preserves resource density while changing the number of regions. Table 10 shows a mild increase in unmet demand and activated capacity on the larger map. The operating pattern remains consistent over the tested range, although the result does not imply size-independent performance.

## 6. Conclusions and Discussion

This study formulates emergency capacity planning as a joint decision over total activation and regional capacity upper bounds. SCGP-RL implements this decision through a compact scarcity-conditioned policy, coupled scalar and capped-simplex projections, and a final feasibility filter. Under the common constraint interface, the method improves urgent-demand service while preserving the planning power margin and executed feasibility. The component results identify the urgent-demand gate as the main response pathway, and the binding-condition tests verify that the power-margin, backhaul, and node-health mechanisms act when their corresponding limits become restrictive.

The results also clarify the role of the proposed design. Its advantage comes from the task-specific factorization of activation amount and regional upper-bound shape before projection. This structure allows the policy to respond to urgent demand without treating all feasible capacity as equally useful, and the robustness and sensitivity studies show that this operating principle persists across the tested conditions. The present validation uses a planning-layer simulator and a calibratable PCU interface. Practical deployment requires technology-specific calibration from bandwidth, spectral efficiency, backhaul availability, power limits, and service measurements. Future work will connect the planning action to LTE or NR scheduling, IAB control, and aerial-platform energy models and will study dynamic regional graphs and online adaptation under new disruption patterns.

## Figures and Tables

**Table 1:** PPO and Projected CPO implementation settings.

Item	Setting
Observation/executed action	3811 state entries; common executed $\left(B_{t},x_{t}\right)$ with N=270
SCGP raw action/network	9 controls; actor 3811–256–256–9, reward critic 3811–256–256–1; tanh
Projected CPO raw action/network	271 controls (one scalar + 270 logits); actor 3811–256–256–271; separate 256–256 reward and cost critics; tanh
Trainable parameters	SCGP-RL 2,085,907; Projected CPO 3,195,424
Training budget	120,000 environment steps; four parallel environments
Learning rate	Linear schedule, $2\times 10^{-4}\to 0$
PPO rollout/batch/epochs	256 per environment/128/10
PPO discount/GAE	γ=0.99/$\lambda_{\mathrm{GAE}}=0.95$
PPO clip/target KL	0.20/0.03
Entropy/value coefficients	0.001/0.5
Maximum gradient norm	0.5
CPO rollout/critic batch	800 per environment (10 complete 80-step episodes); 3200 samples total; batch 400
CPO cost/update	Mean cost budget 0.45; $\gamma_{c}=1.0$; cost GAE 0.95; critic learning rate $2\times 10^{-4}$
CPO trust region	target KL 0.01; CG max 15; damping 0.10; line-search shrink 0.80/max 10; 10 reward- and 10 cost-critic passes
CPO initialization	scalar bias logit(0.10)=−2.1972246; regional-logit biases 0; output weights scaled by 0.10
Checkpoint evaluation interval	5000 environment steps
Structured-prior warm-up	60,000 environment steps
Evaluation action	Deterministic Gaussian mean followed by deterministic decoder
Reward and hard thresholds	Equation (22); all hard residual thresholds equal zero

**Table 2:** Reproducibility and computational measurements.

Item	Setting
Software provenance	Python 3.11.10; NumPy 2.1.2; Pandas 3.0.2; Gymnasium 1.2.3; Stable-Baselines3/SB3-Contrib 2.8.0; PyTorch 2.4.0+cu121; Linux 5.4
Environment split	Train 0–4; validation 1101–1110; test 2101–2130
Training environment seeds	For outer seed *s*, vector environments use s+{0,1009,2018,3027}
Hardware	Dual-socket AMD EPYC 7542 host (128 logical CPUs, approximately 2.0 TiB RAM); NVIDIA GeForce RTX 3090, 24,576 MiB, driver 535.216.03; all training on physical GPU 0
Model records	Architecture-derived parameter counts; checkpoint SHA-256 values and measured file sizes recorded in the run manifests
Hardware platform	AMD EPYC 7542 32-Core Processor; 64 physical/128 logical CPU cores; 2004 GiB host RAM; NVIDIA GeForce RTX 3090 (24 GiB), driver 535.216.03; Python 3.11.10 (main, 7 September 2024, 18:35:41) [GCC 11.4.0], PyTorch 2.4.0+cu121
Training time and memory	120,000 steps in 48.24 min; throughput 41.46 step/s; peak GPU-memory increment 383.0 MiB
Batch-one deployment profile	Policy inference 0.425 ms on average and 0.441 ms at p95; complete prediction and decoding 14.617 ms on average and 14.833 ms at p95; peak RSS 795.4 MiB; peak CUDA allocation 33.7 MiB
Source code	GitHub repository (v1.0.0) https://github.com/MurrayMa0816/SCGP-RL/tree/v1.0.0 (accessed on 16 August 2026)

**Table 3:** Parameters of the nine stress scenarios.

Scenario	Energy Process	$E_{0}$	$E_{\min}$	*H* Mean/Std	High-Priority Demand Setting
Planned charging	deterministic_wave	12.0	2.0	3.0/1.0	base
Random recharge	stochastic	12.0	2.0	3.0/1.0	base
Power outage	outage	12.0	2.0	3.0/1.0	base
Regime shift	regime_shift	12.0	2.0	state vector	base
Low initial energy	outage	9.0	2.0	2.6/1.0	base
High energy floor	regime_shift	12.0	4.0	state vector	base
Urgent-demand surge	outage	11.0	3.0	2.5/1.1	min 0.72/width 0.032
Backhaul capacity contraction	regime_shift	12.0	3.0	state vector	min 0.74/width 0.026
Spectrum contraction/node degradation	stochastic	8.5	3.0	2.4/1.3	min 0.76/width 0.030

**Table 4:** Overall performance in the common held-out environments.

Type	Method	Unmet Score	Backlog	Remaining Power	Activated PCU	Low Power Rate
Main	SCGP-RL	0.492	0.866	15.959	2.559	0.013
Learn.	Fixed-Budget PPO	0.504	0.881	5.886	2.670	1.000
Learn.	Direct-Proj. PPO	0.521	0.916	19.692	2.448	0.012
Learn.	Conservative-Proj. PPO	0.522	0.917	19.916	2.430	0.013
Learn.	Projected CPO	0.664	0.963	21.472	2.091	0.007
Rule	Power Margin Guard	0.573	0.945	13.206	2.619	0.414
Rule	Demand-Gated Act.	0.510	0.898	13.567	2.600	0.082
Rule	Power-Urgent Bound	0.508	0.895	13.183	2.599	0.478
Rule	Power-Region Bound	0.528	0.920	13.175	2.592	0.490
Rule	Rolling-Marginal	0.515	0.901	13.148	2.607	0.128
Rule	Lyapunov DPP	0.515	0.901	13.023	2.608	0.295
Rule	Robust Power Guard	0.585	0.951	14.789	2.606	0.055
Rule	High-Margin Guard	0.617	0.960	17.120	2.588	0.000
Rule	Fixed-Act. Rule	0.587	0.942	5.889	2.711	1.000
Rule	Full-Act. Rule	0.581	0.939	5.867	2.708	1.000
Rule	Uniform Bound Rule	0.646	0.960	5.867	2.732	1.000

Values are means over the common held-out evaluation set.

**Table 5:** Paired comparisons between SCGP-RL and the principal baselines.

Comparator to SCGP-RL	Metric	Effect (95% CI)	Raw *p*	Holm *p*
Projected CPO	Unmet urgent-demand cost	+0.172[+0.167,+0.178]	<0.001	<0.001
Direct-Projected PPO	Unmet urgent-demand cost	+0.029[+0.027,+0.033]	<0.001	<0.001
Rolling-Marginal Greedy	Unmet urgent-demand cost	+0.023[+0.022,+0.024]	<0.001	<0.001

Positive effects favor SCGP-RL. The reported *p*-values use paired tests with Holm correction.

**Table 6:** Effects of component removal relative to complete SCGP-RL.

Removal	Δ Unmet Score	Δ Loss Exceed.	Δ Low Power Rate	Δ Infeasible Proposal	Δ Executed/Proposed	Δ Relief Gain
w/o Power Margin Planning	+0.014	+0.015	+0.019	+0.000	−0.000	−0.009
w/o Urgent-Demand Gate	+0.000	+0.000	+0.000	+0.000	−0.000	+0.000
w/o Marginal-Relief Probe	−0.000	−0.000	−0.000	−0.000	+0.000	−0.000
w/o Urgency Floor	−0.000	−0.000	−0.000	−0.000	+0.000	−0.000
w/o Planning Backhaul Cap	−0.000	−0.000	−0.000	+0.006	−0.000	−0.000
w/o Node-Health Awareness	−0.000	−0.001	+0.000	+0.000	−0.000	−0.000

**Table 7:** Immediate effects of disabling individual components.

Disabled Component	Δ Unmet Score	Δ Loss Exceedance	Δ Demand-Relief Gain
Urgent-demand gate	0.08190	0.16877	−0.06642
Marginal demand-relief estimate	0.00543	0.00725	−0.00295
Urgency safeguard	0.00000	0.00000	0.00000

**Table 8:** Performance across nine resource and demand stress scenarios.

Method	Service-Window Loss	Backlog	Low Power Rate	Relief Ratio	Safe Scenarios
SCGP-RL	0.361	0.878	0.017	98.8%	100%
Projected CPO	0.719	0.963	0.073	61.5%	56%
Conservative-Projection PPO	0.407	0.920	0.066	94.7%	44%
Direct-Projected PPO	0.408	0.920	0.076	94.6%	44%
Fixed-Budget PPO	0.386	0.886	0.986	100.0%	0%
Demand-Gated Activation	0.391	0.904	0.100	98.0%	44%
Power Margin Guard	0.524	0.946	0.399	92.1%	0%
Robust Power Guard	0.550	0.952	0.114	89.7%	22%
Full-Activation Rule	0.548	0.940	0.985	92.1%	0%
Uniform Bound Rule	0.691	0.960	0.985	76.9%	0%

**Table 9:** One-factor-at-a-time sensitivity results.

Setting	Method	Unmet-Demand Cost	Low Power Rate	Mean Activated PCU	Cumulative Service Gain
Canonical control	SCGP-RL	0.492	0.013	2.559	0.142
	Projected CPO	0.664	0.007	2.091	0.085
	Direct-Projected PPO	0.521	0.012	2.448	0.137
$E_{0}/E_{\max}=0.35$	SCGP-RL	0.495	0.025	2.535	0.141
	Projected CPO	0.665	0.024	2.071	0.085
	Direct-Projected PPO	0.523	0.030	2.426	0.136
$E_{0}/E_{\max}=0.525$	SCGP-RL	0.489	0.011	2.583	0.143
	Projected CPO	0.664	0.004	2.109	0.086
	Direct-Projected PPO	0.519	0.008	2.470	0.138
$E_{\min}/E_{\max}=0.08$	SCGP-RL	0.491	0.012	2.571	0.143
	Projected CPO	0.664	0.002	2.091	0.085
	Direct-Projected PPO	0.521	0.005	2.448	0.137
$E_{\min}/E_{\max}=0.17$	SCGP-RL	0.494	0.013	2.546	0.142
	Projected CPO	0.664	0.031	2.091	0.085
	Direct-Projected PPO	0.521	0.040	2.448	0.137
Horizon T=60	SCGP-RL	0.482	0.014	2.503	0.104
	Projected CPO	0.646	0.010	2.052	0.062
	Direct-Projected PPO	0.511	0.012	2.347	0.098
Horizon T=100	SCGP-RL	0.499	0.011	2.570	0.180
	Projected CPO	0.675	0.005	2.107	0.109
	Direct-Projected PPO	0.528	0.020	2.488	0.175
Activation cap =6 PCU	SCGP-RL	0.491	0.014	2.555	0.142
	Projected CPO	0.664	0.002	2.090	0.085
	Direct-Projected PPO	0.519	0.006	2.503	0.139
Activation cap =10 PCU	SCGP-RL	0.494	0.013	2.539	0.141
	Projected CPO	0.664	0.038	2.091	0.085
	Direct-Projected PPO	0.524	0.041	2.392	0.135
Regime replenishment scale =0.80	SCGP-RL	0.520	0.012	2.032	0.118
	Projected CPO	0.668	0.073	1.887	0.077
	Direct-Projected PPO	0.539	0.120	2.033	0.118
Regime replenishment scale =1.20	SCGP-RL	0.463	0.011	3.083	0.165
	Projected CPO	0.663	0.002	2.181	0.089
	Direct-Projected PPO	0.508	0.003	2.775	0.151

Each setting changes one benchmark factor. Cumulative service gain is interpreted together with the episode horizon.

**Table 10:** SCGP-RL sensitivity to planning-map size.

Planning Map	Unmet-Demand Cost	Low Power Rate	Mean Activated PCU	Cumulative Service Gain
12 × 15	0.483	0.007	1.701	0.141
15 × 18	0.492	0.013	2.559	0.142
18 × 21	0.499	0.008	3.568	0.143

Total resources scale with the number of regions to preserve resource density.

## Data Availability

The source code is publicly available at https://github.com/MurrayMa0816/SCGP-RL/tree/v1.0.0 (accessed on 16 August 2026).
